# Changes in Plasma TPH2, GDNF, Trk-b, BDNF, and proBDNF in People Who Died by Suicide

**DOI:** 10.3390/brainsci13071096

**Published:** 2023-07-20

**Authors:** Xiaoyu Liu, Shangda Li, Yueran Yu, Jianbo Hu, Yi Xu

**Affiliations:** 1 Department of Psychiatry, The First Affiliated Hospital, Zhejiang University School of Medicine, Hangzhou 310003, China; 2 Department of Infectious Diseases, Tongde Hospital of Zhejiang Province, Hangzhou 310003, China; 3 The Key Laboratory of Mental Disorder Management in Zhejiang Province, Hangzhou 310003, China; 4 Brain Research Institute of Zijingang Campus of Zhejiang University, Hangzhou 310003, China; 5 Zhejiang Engineering Center for Mathematical Mental Health, Hangzhou 310003, China

**Keywords:** neurotrophic factor, suicide, suicide causes, suicide method

## Abstract

Recent studies have shown that neuropeptides and neurotrophic factors may be involved in the pathophysiological mechanisms of suicide. However, the current research on this aspect is still insufficient. Our study aimed to explore the biological patterns of suicide deaths, including levels of BDNF, proBDNF, BDNF/proBDNF, Trk-b, GDNF, and TPH2. The researchers selected 25 normal control patients matched by age with 30 suicide deaths. We used enzyme-linked immunosorbent assays to detect the levels of BDNF, proBDNF, BDNF/proBDNF, Trk-b, GDNF, and TPH2 in the plasma of suicide and control subjects. proBDNF, BDNF/proBDNF, Trk-b, GDNF, and TPH2 levels are shown as the median (25th–75th percentile). BDNF levels are shown as the mean (standard error of the mean). (1) The levels of plasma TPH2 and proBDNF in people who died by suicide were significantly higher than those in the control group. (2) The plasma levels of GDNF and BDNF/proBDNF in the suicide group were obviously lower than those in the control group. (3) There was no significant difference in plasma BDNF or Trk-b concentrations between the suicide group and the control group.Plasma TPH2, GDNF, and proBDNF levels are related to suicide. Plasma neurotrophic factor markers may predict suicide risk.

## 1. Introduction

According to the World Health Organization, more than 700,000 people worldwide die from suicide every year. Suicide is the fourth-leading cause of death among people aged 15 to 29 [1]. Suicide is a complex phenomenon that is influenced by many social, biological, and psychological factors and their interactions [2]. The suicide methods most commonly used are hanging, pesticide poisoning, and guns. Most suicides are related to mental illness [2,3]. Among these, depression, drug use, and psychosis are the most relevant risk factors [3].

Currently, although many scales have been used to assess suicide risk, biological markers to evaluate suicide risk are still lacking. Research has shown that blood lipid levels may be a biological marker of suicide. Metabolic disorders, especially low total cholesterol and low-density lipoprotein cholesterol levels, may lead to a higher risk of suicide in patients with these mental illnesses [4]. The rs1800532 polymorphism of the SLC6A4 gene encoding the 5-hydroxytryptamine transporter may be associated with susceptibility to suicide [1]. As a result, the biological mechanisms behind suicide have become a recent research hotspot, including the identification of potential biomarkers to assess suicide risk.

Serotonin (5-hydroxytryptamine; 5-HT) is a neurotransmitter in the central nervous system that participates in cognitive activities. It is associated with mental disorders such as depression, autism, schizophrenia, and neurodevelopmental disorders [5]. Previous studies have suggested a link between serotonin dysfunction in the brain and suicidality [6]. Tryptophan hydroxylase (TPH) is the rate-limiting enzyme of 5-HT and has two forms, TPH1 and TPH2 [7]. TPH2 is abundant in the central nervous system, while TPH1 is highly expressed in intestinal chromaffin cells [8]. TPH2 is most abundantly expressed in the dorsal raphe nuclei (DRN) [9]. The human TPH2 gene is located on chromosome 12q15, comprises 11 exons, and covers a region of 93.5 kb [10].

Neurotrophic factors are involved in the growth, differentiation, and death of neurons as well as inflammation [11,12,13]. Glial cell-derived neurotrophic factor (GDNF) is a 134-amino acid protein [14]. Currently, GDNF is believed to be a necessary factor in the development, maintenance, and protection of DA neurons in the black striatum, as well as a potential factor involved in the protection and recovery of affected DA neurons in Parkinson’s disease [15,16]. The GDNF protein is widely distributed in the CNS and PNS [14]. GDNF is considered a major regulator of the DA system and, in addition to BDNF, interacts with 5-HT in the brain [17]. An increasing number of studies have shown that GDNF is also involved in emotion regulation [18]. Recently, research has found a relationship between BDNF and GDNF and the occurrence of mental illness. Studies have found that a decrease in plasma BDNF and GDNF levels is a powerful indicator for predicting the occurrence of MDD [19].

Brain-derived neurotrophic factor (BDNF) is a protein composed of 247 amino acids [20]. It is also one of the most studied and widely distributed neurotrophic factors in vivo and plays a very important role in brain function and neuroprotection [21,22]. A previous study found that the expression of BDNF was significantly decreased in patients with depression [23]. Furthermore, animal studies have shown that chronic unpredictable stimuli significantly reduce hippocampal BDNF mRNA levels [24,25]. The precursor of BDNF is proBDNF, which is synthesized in neurons and glial cells. It is cleaved into BDNF inside or outside the cell [26]. BDNF and proBDNF have opposite functions, and their roles are mediated by tropomyosin-associated kinase B (Trk-b) receptors and nonspecific p75 receptor 2, respectively [17]. BDNF induces axon growth and dendritic maturation [27]. In contrast, proBDNF inhibits neurite outgrowth [28]. A study also found that environmental factors are important regulatory factors in the balance between proBDNF and BDNF, playing an important role in their transformation [29]. Trk-b has been found to be involved in the neurobiology of suicide [30]. Some studies have shown that the level of BDNF in the hippocampus of mice with tryptophan hydroxylase (Tph2) deficiency was significantly increased, which indicated that the level of BDNF was affected by the amount of TPH2 expression [31]. Trk-b is a single-channel type 1 membrane protein that contains a protein kinase domain, two leucine-rich repeats, and two Ig-like C2 group domains [32]. Trk-b is expressed in both the central nervous system (CNS) and peripheral nervous system (PNS) [32]. A study has shown that both BDNF and its receptor, Trk-b, are downregulated in the prefrontal cortex and hippocampus of adolescent suicide victims [33].

Our hypothesis is that 5-HT expression decreases in individuals who commit suicide. This leads to a reduction in the expression of the nutritional factors BDNF and GDNF, which may be related to an increase in TPH2 expression. This may induce impulsive suicidal behavior. In the present study, we explored suicide-related biomarkers for treatment and prevention. We measured plasma levels of BDNF, proBDNF, BDNF/proBDNF, Trk-b, GDNF, and TPH2 in people who died by suicide and in age- and sex-matched healthy controls. In addition, we analyzed changes in TPH2, BDNF, and GDNF in mental illness suicide (PI) and life event suicide (LE). We suggest that plasma TPH2, BDNF, and GDNF might be potential biomarkers for suicide.

## 2. Materials and Methods

### 2.1. Characteristics of Suicide and Healthy Control Subjects

Plasma samples were collected from 30 suicide subjects (21 males and 9 females) in the Hangzhou Public Criminal Investigation Detachment of Public Security Bureau, and they were matched for gender and age with 25 healthy controls (HCs). Informed consent for the plasma material and medical records for research purposes was given by the suicide subjects’ next of kin and the healthy control subjects.

The suicide subjects matched well with the control group for age (Table 1, *p* = 0.993) and gender (see Table 1, *p* = 0.1). In this experiment, psychiatric illness (PI) and life events (LE) were the two factors that induced suicide. Of the 30 suicide subjects, PIs included schizophrenia and major depressive disorders, while LEs were related to health issues, family issues, and social issues (Table 2). Ten suicide subjects had PIs, and their deaths were associated with irregular medication use, while the remaining 20 suicide subjects experienced LEs associated with various cultural backgrounds, such as religion, quarrels, and organic disease.

In addition, suicide deaths were also divided into violent or nonviolent suicide attempts [34,35]. Nonviolent suicide attempts included drug overdoses (4, 13.3%) or a single wrist cut. Violent attempts included jumping (11, 36.6%), hanging (8, 26.7%), drowning (2, 6.7%), and carbon monoxide poisoning (5, 16.7%) (Table 2).

### 2.2. Acquisition of Plasma Samples from Suicide Subjects

The conditions of the control group were as follows: 1. Age and gender were matched with suicide subjects. 2. Subjects had no lifetime history of serious physical illness, organic brain disease, or brain injury. 3. There was no severe mental retardation, and MRI showed no structural abnormalities in the brain. 4. No subjects had metal in their bodies, such as pacemakers, insulin pumps, or artificial heart valves. Twenty-five healthy control subjects met the inclusion criteria. This study was approved by the ethics committee of the First Affiliated Hospital of Zhejiang University School of Medicine and registered in the China Clinical Trial Registry (number ChiCTR 180015937).

### 2.3. Blood Collection Method

Venous blood samples were collected using anticoagulant tubes. The blood samples were centrifuged at 3000 rpm for 20 min at 4 °C. Finally, the samples were stored at −80 °C until the assay was performed.

### 2.4. Serum Peptide Concentration Measurement

Enzyme-linked immunosorbent assay (ELISA) was used to determine the plasma levels of BDNF, Trk-b, proBDNF, GDNF, and TPH2. Plasma concentrations of BDNF (Cloud-Clone, Wuhan, China), Trk-b (Cloud-Clone, Wuhan, China), proBDNF (DY3175 R&D, Minneapolis, MN, USA), TPH2 (SEG343Hu, Cloud-Clone, Wuhan, China), and GDNF (SEA043Hu, Cloud-Clone, Wuhan, China) were measured by ELISA kits according to the manufacturer’s notes. All experiments were performed in duplicate.

### 2.5. Statistical Analysis

The data are presented as the median (25–75%) and were analyzed with SPSS 19.0 (IBM, Chicago, IL, USA). Since biological data were not normally distributed except for BDNF, nonparametric tests were used for proBDNF, GDNF, Trk-b, and TPH2. Kruskal–Wallis (K-W) tests were used to determine the differences among the three groups. If a significant difference was found, the Mann–Whitney U test was used to evaluate the difference between the two groups. BDNF was normally distributed, as determined using an independent-samples *t*-test. Spearman’s test was used for correlation analysis. All tests were double-tailed, with *p* values ≤ 0.05 considered significant, while *p* values < 0.1 and >0.05 were considered trends. Binary regression analyses were conducted to measure the specificity and interaction of BDNF, proBDNF, GDNF, Trk-b, and TPH2 on suicide risk.

## 3. Results

Changes in plasma TPH2 levels between healthy control subjects and suicide subjects.

**Figure 1:**
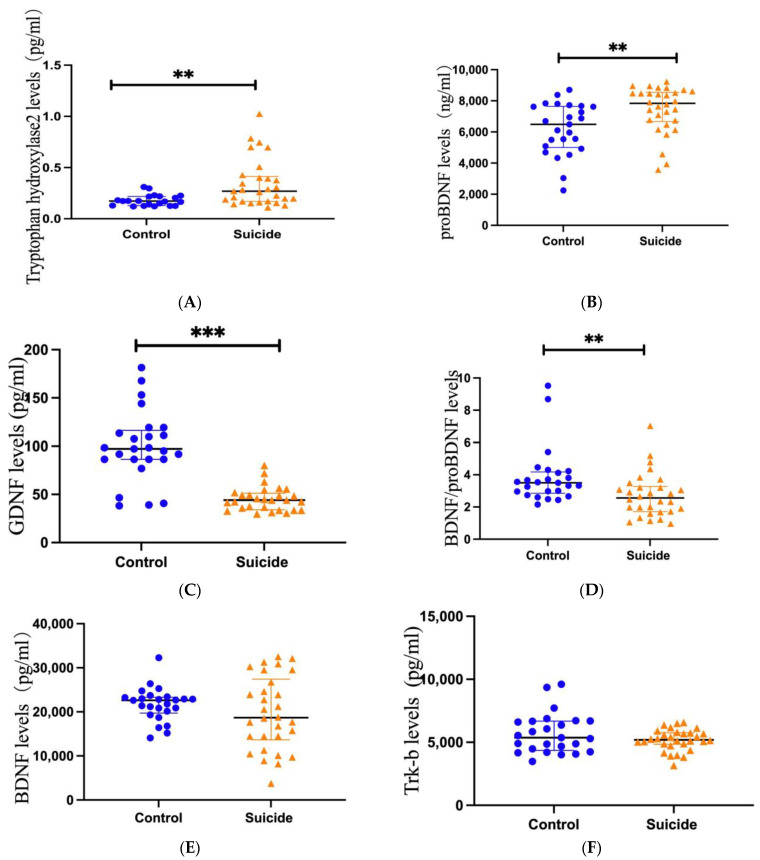
Scatter dot plots showing plasma levels of TPH2, proBDNF, GDNF, BDNF/proBDNF, BDNF, and Trk-b between suicide (*n* = 30) and controls (*n* = 25). Changes in plasma levels of TPH2 (**A**), proBDNF (**B**), GDNF (**C**), BDNF/proBDNF (**D**), BDNF (**E**), and Trk-b (**F**) from suicides (right) and controls (left). Blue plots represent controls and yellow triangles represent suicides. Significance levels are indicated as ** *p* < 0.01, *** *p* < 0.001.

**Figure 2:**
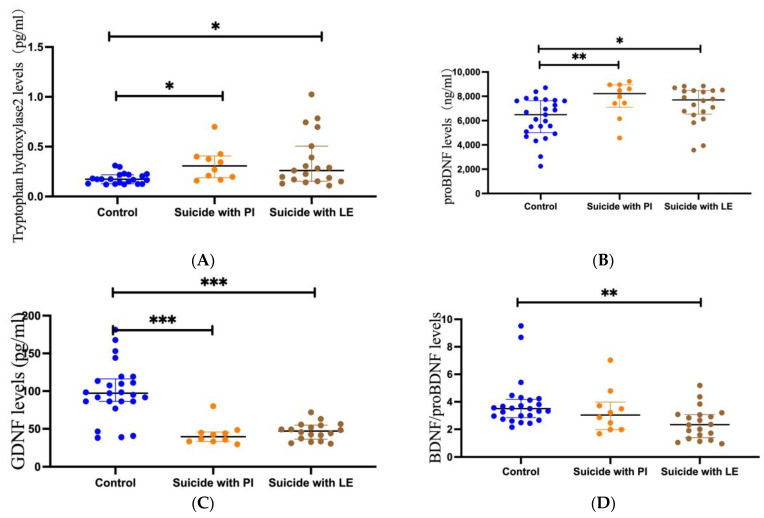
Scatter dot plots showing plasma levels of TPH2, proBDNF, GDNF, and BDNF/proBDNF between suicides with life events (LE) (*n* = 20), suicides with psychiatric illness (PI) (*n* = 10), and controls (*n* = 25). Changes in plasma levels of TPH2 (**A**), proBDNF (**B**), GDNF (**C**), and BDNF/proBDNF (**D**) in suicides with life events (LE) (right), suicides with psychiatric illness (PI) (medial), and controls (left). Blue plots represent controls, yellow plots represent suicides with psychiatric illness (PI), and brown plots represent suicides with life events (LE). Significance levels are indicated as * *p* < 0.05, ** *p* < 0.01, *** *p* < 0.001.

The Mann–Whitney U test showed that plasma TPH2 concentrations were significantly higher in suicide subjects than in healthy controls (*p* = 0.003, Figure 1A). Additionally, the Mann–Whitney U test showed that the plasma TPH2 concentration was significantly higher in suicide subjects with PIs than in controls (*p* = 0.004, Figure 2A). Similarly, the concentration of TPH2 in subjects with LEs was significantly higher than that in the control group (*p* = 0.017, Figure 2A). However, no significant difference was found between PI and LE suicides (*p* = 0.636, Figure 2A). The Mann–Whitney U test showed that no significant difference was found in TPH2 concentration between violent and nonviolent suicide subjects (*p* = 0.341).

### 3.1. Changes in Plasma Levels of proBDNF between Suicide and Control Subjects

The Mann–Whitney U test showed that the plasma levels of proBDNF in the suicide subjects were obviously lower than those in the plasma of healthy control subjects (*p* = 0.003, Figure 1B). The Mann–Whitney U test also showed that the plasma proBDNF concentrations in PI suicide subjects (*p* = 0.008) and LE suicide subjects (*p* = 0.016) were significantly lower than those in the control group, while the Mann–Whitney U test showed no significant difference between PI suicide subjects and LE suicide subjects (*p* = 0.214, Figure 2B). In addition, the Mann–Whitney U test showed that there was no difference in proBDNF levels between violent and nonviolent suicide attempts (*p* = 1).

### 3.2. Changes in Plasma Levels of GDNF between Suicide and Control Subjects

The Mann–Whitney U test showed that GDNF levels in the plasma of suicide subjects were significantly lower than those in control subjects (*p* < 0.001, Figure 1C). The Mann–Whitney U test also showed that the plasma GDNF concentrations in PI suicide subjects (*p* < 0.001) and LE suicide subjects (*p* < 0.001) were obviously lower than those in the healthy control group, while the Mann–Whitney U test showed no significant difference between PI suicide subjects and LE suicide subjects (*p* = 0.226, Figure 2C). Furthermore, a *t*-test found no difference in GDNF levels between violent and nonviolent suicide attempts (*p* = 0.924).

### 3.3. Changes in Plasma Levels of BDNF/proBDNF between Suicide and Control Subjects

The Mann–Whitney U test showed that BDNF/proBDNF concentrations in the plasma of suicide subjects were obviously lower than those in healthy controls (*p* = 0.003, Figure 1D). In addition, the Mann–Whitney U test showed no difference between the PI and control groups (*p* = 0.225, Figure 2D). The Mann–Whitney U test showed that the concentration of BDNF/proBDNF in subjects with LE was significantly lower than that in the control group (*p* = 0.001, Figure 2D). However, the Mann–Whitney U test showed no significant difference between PI suicide subjects and LE suicide subjects (*p* = 0.12, Figure 2D). The Mann–Whitney U test showed that no significant difference was found in BDNF/proBDNF concentrations between violent and nonviolent suicide attempts (*p* = 0.123).

### 3.4. Changes in Plasma Levels of BDNF between Suicide and Control Subjects

An independent-samples *t*-test showed no significant difference in BDNF concentrations between control and suicide subjects (*p* = 0.238, Figure 1E). An independent-samples *t*-test showed no significant difference between PI suicide subjects and LE suicide subjects (*p* = 0.045). In addition, Mann–Whitney U test showed that no significant difference was found in BDNF concentrations between violent and nonviolent suicide attempts (*p* = 0.094).

### 3.5. Changes in Plasma Levels of Trk-b between Suicide and Control Subjects

The Mann–Whitney U test showed no significant difference in Trk-b levels between control and suicide subjects (*p* = 0.357, Figure 1F). The independent samples test also showed no difference in Trk-b concentration between those who died of violent and nonviolent suicide attempts (*p* = 0.359).

### 3.6. Correlations between Different Serum Peptide Levels and General Information

There were no significant correlations among TPH2, proBDNF, GDNF, Trk-b, BDNF levels and age in suicide subjects (all *p* > 0.07) or control subjects (all *p* > 0.1).

### 3.7. Hierarchical Regression Analysis of BDNF, proBDNF, GDNF, Trk-b, and TPH2 on Suicide Risk

Binary regression analysis showed no significant difference in BDNF, proBDNF, GDNF, Trk-b, or TPH2 levels between the control group and the suicide subjects (all *p* ≥ 0.126). Binomial regression analysis showed no statistically significant differences in BDNF, proBDNF, GDNF, Trk-b, and TPH2 levels in PI suicides and LE suicide subjects (all *p* ≥ 0.103).

## 4. Discussion

In this study, plasma levels of TPH2, proBDNF, GDNF, and BDNF/proBDNF were shown to be associated with suicide. The plasma concentrations of TPH2 and proBDNF in the suicide group were higher, while the plasma concentrations of GDNF and the ratio of BDNF/proBDNF were lower. In addition, suicidal patients with psychiatric disorders and relevant life events had higher plasma concentrations of TPH2 and proBDNF than healthy controls. In contrast, plasma concentrations of GDNF and BDNF/proBDNF were reduced in individuals with psychiatric disorders and those with suicides caused by life events. Studies have shown that plasma TPH2, proBDNF, GDNF, and BDNF/proBDNF concentrations are associated with suicide but not with the cause or manner of suicide. Therefore, according to the above results, TPH2, the BDNF-Trk-b pathway, and GDNF in plasma are involved in the process of human death by suicide.

The present experiment showed that plasma TPH2 levels were upregulated in suicidal individuals, which may indicate that TPH2 is involved in regulating the pathophysiological process of suicide. Previous studies have found that suicide can occur in people with psychiatric disorders, such as depression and personality disorders. Adverse life events and stress affect the function of the brainstem serotonergic system, leading to affective and anxiety disorders [36,37]. Platelet 5-HT concentrations were found to be significantly lower in patients with suicidal tendencies at the first episode of psychosis than in nonsuicidal patients and healthy controls [38]. Postmortem examination showed that prefrontal cortex levels of 5-hydroxytryptamine and its metabolites were decreased in patients who died by suicide, suggesting that suicide is related to the decreased function of central serotonin [39]. As a key rate-limiting enzyme for 5-HT synthesis, the compensatory increase in TPH-2 may be involved in the regulation of suicide when the level of 5-HT is decreased. Donner and his colleagues also found in animal studies that acute stress increased TPH activity in the dorsal raphe nucleus in male rats, and chronic binding stress increased TPH activity in the dorsal raphe nucleus, which is consistent with the present findings [40]. We suggest that the increased TPH levels might be due to the hypoactivity of 5-HT, which may indicate an elevated suicide risk.

ProBDNF levels were significantly increased in the plasma of suicidal individuals. ProBDNF may be related to the increase in depression. Stress events such as chronic stimulation and acute stress increase the level of proBDNF. It is now generally believed that BDNF and proBDNF have opposite effects. It has been reported that proBDNF induces neuronal atrophy, apoptosis, and dendritic pruning [41]. This may explain the elevated plasma proBDNF concentration in suicide subjects.

The BDNF/proBDNF ratio was significantly decreased in the plasma of suicide subjects, but there was no significant difference in BDNF, indicating that proBDNF was increased under stress conditions. These results are similar to those of an animal study that reported that elevated levels of proBDNF were found in the hippocampus of an animal model of stress depression, resulting in a decreased BDNF/proBDNF ratio [41,42]. It can be hypothesized that increased expression of proBDNF and a decreased BDNF/proBDNF ratio lead to an imbalance between BDNF and its precursor, which contributes significantly to the occurrence of suicide. However, some studies have shown that serum levels of GDNF, NGF, and NTF3 in patients with major depression are not significantly different. Neurotrophic levels were not associated with suicidal ideation or suicide. Ayhan Bilgiç’s team also found that levels of nutritional factors, including BDNF, were not associated with suicidal ideation and behavior [43].

The plasma level of GDNF was significantly lower in the suicide group. Furthermore, as 5-HT can promote the expression of GDNF [44], low GDNF levels may suggest an insufficient function of 5-HT. On the basis of the reduction in GDNF caused by life events, chronic stress, and other factors [45], we suggest the reduction in 5-HT levels will lead to a decrease in GDNF expression. Therefore, the downregulation of GDNF levels may indicate the risk of suicide. and proBDNF and GDNF acted synergistically to upregulate the expression of TPH2, indicating that the increased expression of TPH2, proBDNF, and GDNF in suicide is reasonable. In addition, it has been reported that GDNF can protect dopaminergic neurons by regulating BDNF through Pitx3 [46].

We did not observe significant changes in plasma BDNF in suicide subjects. Smith et al. found that stress can reduce the expression of BDNF in the hippocampus [47], and BDNF levels are negatively correlated with the severity of depression. Serum BDNF levels in patients with major depression are significantly lower than those in healthy individuals, and BDNF levels in severe patients are lower than those in moderate patients [48]. According to the above results, researchers tried to determine whether there was a correlation between suicide and BDNF, but the results were negative. Liying Lin’s results showed that there was no significant change in serum BDNF in people who had attempted suicide compared with the control group [48]. Eisen R.B. found no significant association between suicide attempts and BDNF levels [49], consistent with our results. Moreover, in our study, six people had depression without depression scores, and four people had mental disorders. There was a lack of correlation between the severity of the mental disorder and serum BDNF concentration. Disruption of the 5-HT-BDNF system interaction can result in depressive disorder, suicidality and aggressiveness [50]. On the other hand, we suggest the concentration of 5-HT was reduced in suicidal individuals, and the effect of BDNF on neurons could increase the amount of 5-HT by increasing the amount of TPH2. In other studies, BDNF mRNA levels in the prefrontal cortex and hippocampus were significantly reduced in subjects who died by suicide. Moreover, when they divided suicidal people into two groups with a history of major depression and a history of other psychiatric disorders, they found no difference in BDNF expression between the two groups, and all suicidal people had decreased BDNF levels regardless of psychiatric diagnosis. Their findings suggest that decreased BDNF levels are associated with suicide [51]. In summary, plasma BDNF is currently more suitable as a biological indicator of depression severity than as a biological indicator of suicidal ideation.

No changes in plasma Trk-b were found in suicidal patients in this study. Trk-b is one of the major receptors to which BDNF binds with high affinity and mediates its action. In this study, the activity of Trk-b may be impaired in suicidal patients, leading to the dysgenesis of BDNF. Studies have shown that full-length Trk-b expression is significantly decreased in the prefrontal cortex and hippocampus of adult suicidal subjects compared with age-matched healthy controls, and Trk-b T1 expression is not significantly changed. When the brains of suicidal adolescent subjects were examined, similar changes in Trk-b expression were found at full length. This may be related to the location of the specimen.

## 5. Limitations

The control group was composed of healthy people, not healthy people who died accidentally. The sample size was relatively small due to the heterogeneity of the causes of death. In this study, 63 suicide samples were collected, and the remaining 33 samples were not used because of a lack of identity information or a lack of PI or LE, which were unknown causes of death. In the future, western blotting and other confirmatory experiments should be performed on brain tissue. The study of plasma TPH2 and BDNF levels in people with suicidal ideation or suicide attempts also needs further research. More advanced methods were used to assess the relative contribution of each biomarker to suicide risk in the suicide group. Distinguishing between PI and LE, we collected patients who had a history of mental illness and had a history of arguments with others during their lifetime, and we divided them into PI and LE based on the information provided by the patient’s family. Due to the small sample size and incomplete detailed information on individuals who died by suicide, our experiment did not specifically classify subjects according to health issues, family factors, or social factors for statistical analysis. Our study did not involve high-risk populations, such as patients with previous suicide attempts, which is also a deficiency in our experimental design.

## Figures and Tables

**Table 1:** General Information and Parameters Measured for Controls and Suicides.

	Controls	Suicides	*p* Value
Age (years)	36 (30–47)	42.27 ± 18.684	0.993
Gender (male/female)	12/13	21/9	0.1
TPH2	0.17 (0.13–0.22)	0.27 (0.17–0.41)	0.003
proBDNF	6490.80 (5006.80–7646.80)	7834.80 (6666.80–8535.80)	0.003
GDNF	97.30 (86.38–116.30)	44.07 (34.15–51.24)	*p* < 0.001
BDNF/proBDNF	3.50 (2.85–4.18)	2.56(1.71–3.28)	0.003
BDNF	21,707.71 ± 3783.93	19,690.00 ± 8246.60	0.238
Trk-b	5364.99 (4359.29–6676.00)	5192.00 (4843.91–5760.98)	0.357

Note: Data are presented as Median (25th–75th); BDNF are presented as Mean ± SD.

**Table 2:** The Distribution of Suicide Methods and Suicide Causes.

		Violent Way	Non-Violent Way
Psychiatric	SC	4	0
Illness	MDD	5	1
Life events	Health Problem	3	1
	Family Problem	3	0
	Social Factor	5	0
	Unknow	6	2

## Data Availability

The data presented in this study are available on request from the corresponding author. The data are not publicly available due to privacy of people who died by suicide.
